# The Use of Adipose Derived Stem Cells in Chronic Wound Healing

**DOI:** 10.1111/iwj.70986

**Published:** 2026-07-13

**Authors:** Siôna M. Ladley, Seyran Naghdi

**Affiliations:** ^1^ Warwick Medical School University of Warwick Coventry UK; ^2^ Warwick Clinical Trials Unit, Warwick Medical School Coventry UK

**Keywords:** adipose derived stem cells, chronic wounds, ulcers, wound healing

## Abstract

Wound healing is a complex process of angiogenesis, inflammation and proliferation relying on co‐ordination of multiple cell types and cytokines. Dysregulation of this process can lead to chronicity, which we are seeing more commonly due to an increasingly comorbid population. Adipose‐Derived Stem Cells (ADSCs) show promise as a therapy though their ability to augment the tissue microenvironment via multiple pathways. This review aims to synthesise evidence on the effects of ADSCs on chronic wound healing to assess their efficacy. A systematic search of four databases was conducted covering a period up to June 2025. Comparative studies investigating ADSCs and wounds of any aetiology were included, and a meta‐analysis was performed on pooled data. Ten studies were included in the meta‐analysis (*N* = 498). Patients treated with ADSCs were more likely to heal at short‐term follow‐up (odds ratio (OR) 6.54, 95% confidence interval (CI) 3.80–11.27; *n* = 377 *I* ^2^ = 0.00% *p* = 0.00) and healed significantly faster (mean difference −1.90, CI −2.28, −1.51; *n* = 210, *I* ^2^ = 23.58, *p* = 0.00) than controls. Grades of recommendation, Assessment, Development and Evaluation assessment showed this evidence is of moderate certainty. This review presents clinical evidence that ADSC use is a safe therapy which expedites the healing of wounds from multiple aetiologies.

## Key Points

- Patients treated with ADSCs healed faster and were more likely to have healed at short‐term follow‐up compared to those receiving standard care.
- Clinical trials included in this meta‐analysis are clinically heterogenous, varying significantly in wound type, ADSC administration method, and cohort comorbidity.
- Further high‐quality clinical trials with standardised treatment protocols are needed to determine a dose‐response relationship.

## 1 Introduction

There are approximately 2.2 million people in the UK living with chronic wounds, costing the National Health Service an estimated £5.5 billion per year [1]. Normal wound healing relies on an orderly progression through four overlapping phases: haemostasis, inflammation, proliferation and resolution [2]. In most chronic wounds, this process halts during the inflammation stage, resulting in chronic inflammation and degradation of the extracellular matrix (ECM) [3]. Chronic wounds are generally defined as wounds that fail to begin healing after 4–12 weeks [4, 5]. Numerous factors can contribute to impaired healing; comorbidity with chronic illnesses is a leading contributor [3, 6]. Conventional treatment for chronic wounds depends on the wound type; however, there are common principles such as aseptic cleaning, appropriate dressing and occasional use of antimicrobial dressings [7]. Current National Institute for Health and Care Excellence (NICE) guidance highlights the lack of robust evidence for the use of these dressings [8], this underscores the urgent need for novel treatments.

Stem cells are unique in their ability to differentiate into multiple cell types and undergo prolonged self‐renewal [3]. Adipose tissue and the stem cells found within it have been investigated in cartilage repair [9, 10] and wound healing [11] to some success. Adipose‐derived stem cells (ADSCs) are a type of mesenchymal stem cell (MSC) capable of differentiating into key cell types involved in wound healing including fibroblasts, keratinocytes, epithelial cells and endothelial cells [3]. Alongside this differentiation, ADSCs augment the healing tissue microenvironment through resistance to stressors such as hypoxia [12], promoting angiogenesis [13], reducing chronic inflammation [14, 15] and secretion of exosomes and ECM‐augmenting matrix metalloproteases [16]. Another benefit of ADSCs is that the harvesting process is less invasive than that for MSCs of other types, for example bone marrow MSCs [16]. These properties make ADSCs particularly suitable for addressing the challenges of chronic wound healing. Wounds that fail to heal within expected timeframes can cause considerable distress to patients [17]. Clinical trials suggest that applying ADSCs to chronic wounds may accelerate the healing process, although many studies lack control groups to confirm their efficacy [18, 19].

This review aims to synthesise data from studies investigating ADSCs' impact on wound healing to determine whether they offer significant effectiveness over the current conventional therapies outlined.

## 2 Materials and Methods

This review is reported following Preferred Reporting Items for Systematic Reviews and Meta‐Analyses (PRISMA) guidelines [20] and registered on the OSF DOI: 10.17605/OSF.IO/X4TP6.

### 2.1 Inclusion and Exclusion Criteria

Studies were included that evaluated the clinical effectiveness of ADSCs in chronic wound healing. Randomised and non‐randomised control trials were included. Chronic wounds of any aetiology were considered, and fat grafts alone were only considered if they quantified the presence of stem cells. Wounds were considered chronic if they had been present for ≥ 3 months. Exclusion criteria were as follows: animal studies, non‐controlled trials, studies on non‐chronic wounds and in vitro studies.

### 2.2 Search Strategy and Selection Criteria

Four databases were systematically searched in October 2024 and June 2025 (Appendix A). Embase and Medline were searched using OVID search strategy using Medical Subject Headings (MeSH) outlined in Appendix B; this was used as a template for the other database searches. The retrieved studies were imported into EndNote [21] where they were screened for duplications using the work of Wichor Bramer [22] as a guide. Rayyan [23] was used to perform further duplication removal and title and abstract screening according to population, intervention, comparison and outcomes criteria. The researcher reviewed full texts of the studies according to the prespecified inclusion/exclusion criteria, consulting with the supervisor if necessary.

### 2.3 Data Extraction

Data extraction was performed by one researcher and verified by the second researcher. The following data was extracted onto an excel spreadsheet [24]: Study title, authors, year of publication, number of participants, patient characteristics, intervention and comparator and outcomes of interest. Means, standard deviations (SD) and proportions of patients in each intervention were extracted for each outcome as appropriate. If SDs were not provided, they were calculated following Cochrane's guidance for meta‐analyses [25].

### 2.4 Outcome Measures

The five clinical outcomes of interest were: 1. Healing achieved, 2. Time taken to heal, 3. Rate of healing, 4. Adverse events and 5. Patient satisfaction.

### 2.5 Follow‐Up Timepoint Categorisation

Outcomes that were based on follow‐up periods were categorised as follows: short‐term (< 12 weeks) and long‐term (> 12 weeks).

### 2.6 Assessment of Risk of Bias and Certainty in Evidence

The researcher performed the quality assessment, consulting with the second researcher as necessary. Bias in non‐randomised studies was assessed using the Risk Of Bias In Non‐randomised Studies—of Interventions (ROBINS‐I) tool [26] and the Revised Cochrane Risk of Bias tool for randomised trials (RoB 2) [27] was used for randomised studies. Grades of Recommendation, Assessment, Development and Evaluation (GRADE) approach was used to assess certainty of evidence [28].

### 2.7 Statistical Analysis

To assess the effectiveness of ADSC use, meta‐analyses using Hedges' random effects model [29] were performed on studies which had outcomes that were appropriately homogenous to facilitate comparison between outcomes. Effect sizes and 95% Confidence Intervals (CIs) were displayed on forest plots. STATA 18 [30] was used to perform all analyses; the *I* ^2^ statistic was used to assess statistical heterogeneity, with values > 50% considered significantly heterogenous [25]. Results were considered statistically significant at *p* < 0.05. Subgroup analyses according to wound type, ADSC source, or delivery method were not performed due to the limited number of studies within each subgroup and substantial methodological heterogeneity, which would likely produce unreliable estimates.

## 3 Results

### 3.1 Study Selection

**FIGURE 1:**
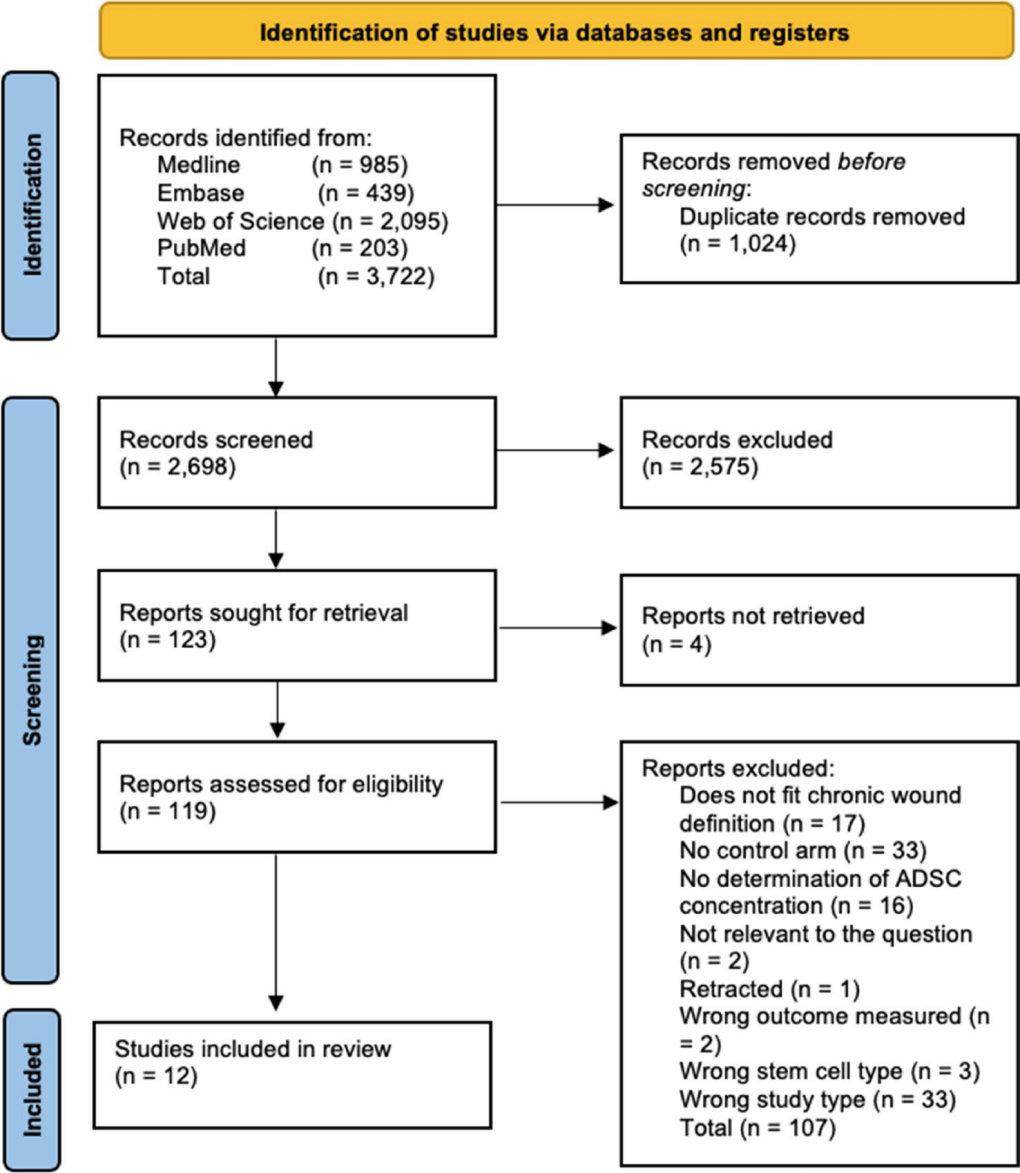
PRISMA diagram. ADSC: Adipose‐Derived Stem Cell; PRISMA: Preferred Reporting items for Systematic reviews and Meta Analyses.

Searches yielded 3722 papers after removal of duplicates. 2575 records were excluded after title and abstract screening, leaving 123 for full‐text screening, four of which were not retrieved (Figure 1). Twelve studies [14, 31, 32, 33, 34, 35, 36, 37, 38, 39, 40, 41] met the inclusion criteria and were included in the analysis.

### 3.2 Study Characteristics

Of the included studies, nine were randomised [14, 31, 34, 36, 37, 38, 39, 40, 41] and three were non‐randomised [32, 33, 35]. Twelve studies involving 519 participants met the inclusion criteria for the systematic review. Of these, 10 studies comprising 498 participants contributed data to the meta‐analyses. Overall patient age ranged from 15 to 89 years. Percentage of females ranged from 0% to 51%. Seven studies [14, 31, 33, 34, 35, 37, 41] reported short‐term outcomes, and four studies [14, 36, 38, 40] reported long‐term outcomes. The most common comorbidity was diabetes mellitus (DM), although not all studies provided complete comorbidity data [14, 31, 36, 37, 39, 41]. There was a large variety of wound types, with diabetic ulcers and perianal fistulae being the most common (Appendix D). All included studies utilised ADSCs; however, there was substantial variability in ADSC preparation, characterisation and concentration. Eight studies [14, 32, 33, 36, 37, 39, 40, 41] included ADSCs which were harvested from the patient before being returned to the same patient (autologous), and three [34, 35, 38] included ADSCs which had been harvested from a donor before administration to a patient in the study (allogenic). One study [31] did not provide information on ADSC source. ADSC delivery methods also varied considerably and included intralesional injection, topical application and dressings. Characterisation techniques were inconsistently reported across studies, ranging from centrifugation alone to flow cytometry and immunophenotyping [32, 33, 34, 35, 37, 40, 41]. Furthermore, only five studies [33, 35, 38, 39, 41] reported detailed information regarding ADSC concentration or cell dose administered. This variability limited comparison between studies and prevented meaningful assessment of dose–response relationships. Patient and ADSC characteristics by study and outcome measured are outlined in Appendices C, D, E.

### 3.3 Risk of Bias Assessment and Certainty in Evidence

Most of the randomised control trials had a high risk of bias [31, 34, 36, 37, 39, 40]. Three studies [31, 34, 37] were single blinded, where the researcher measuring wound healing was aware of which treatment the patient had. Three included studies [32, 33, 35] were non‐randomised; the study which is included in the meta‐analyses [35] has a moderate risk of bias due to lack of blinding. Figures outlining risk of bias assessment for randomised and non‐randomised control trials can be found in Appendices F and G, respectively. Low to moderate certainty in evidence was found and reported in Table 1.

**TABLE 1:** Summary of findings.

Population: Patients with chronic wounds Intervention: ADSC administration Comparison: Conventional treatments (varied)					
Outcomes		Effect size; OR/Hedge's g (95% CI), *p*.	No of patients (studies)	Certainty of evidence (GRADE) [28]	Comments
Complete healing achieved	Short‐term	OR: 6.54 (3.89 to 11.27), *p* = 0.00	377 (6)	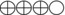	OR > 1 suggests that ADSC treatment increased likelihood of achieving full healing
	Long‐term	OR: 1.76 (0.79 to 3.92), *p* = 0.16	192 (4)	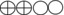	
Time taken to heal		Hedges g: −1.9 (−2.28 to −1.51), *p* = 0.00	210 (5)	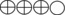	Hedges g < 0 suggests that ADSC treatment decreased the time taken to achieve healing
GRADE Working Group grades of evidence *High certainty:* Further research is very unlikely to change our confidence in the estimate of effect. *Moderate certainty:* Further research is likely to have an important impact on our confidence in the estimate of effect and may change the estimate. *Low certainty:* Further research is very likely to have an important impact on our confidence in the estimate of effect and is likely to change the estimate. *Very low certainty:* Authors of this review are very uncertain about the estimate.					

Abbreviations: ADSC: Adipose Derived Stem Cells, CI: Confidence interval, OR: Odds ratio.

### 3.4 Meta‐Analysis Results

Due to the diverse nature of the studies and differing ways of quantifying healing, only two of the outcomes of interest had enough studies with comparable results to perform a meta‐analysis: healing achieved and time taken to heal (Table 1). Two studies [32, 33] are not included in any meta‐analyses, due to their methodology.

### 3.5 Healing Achieved

**FIGURE 2:**
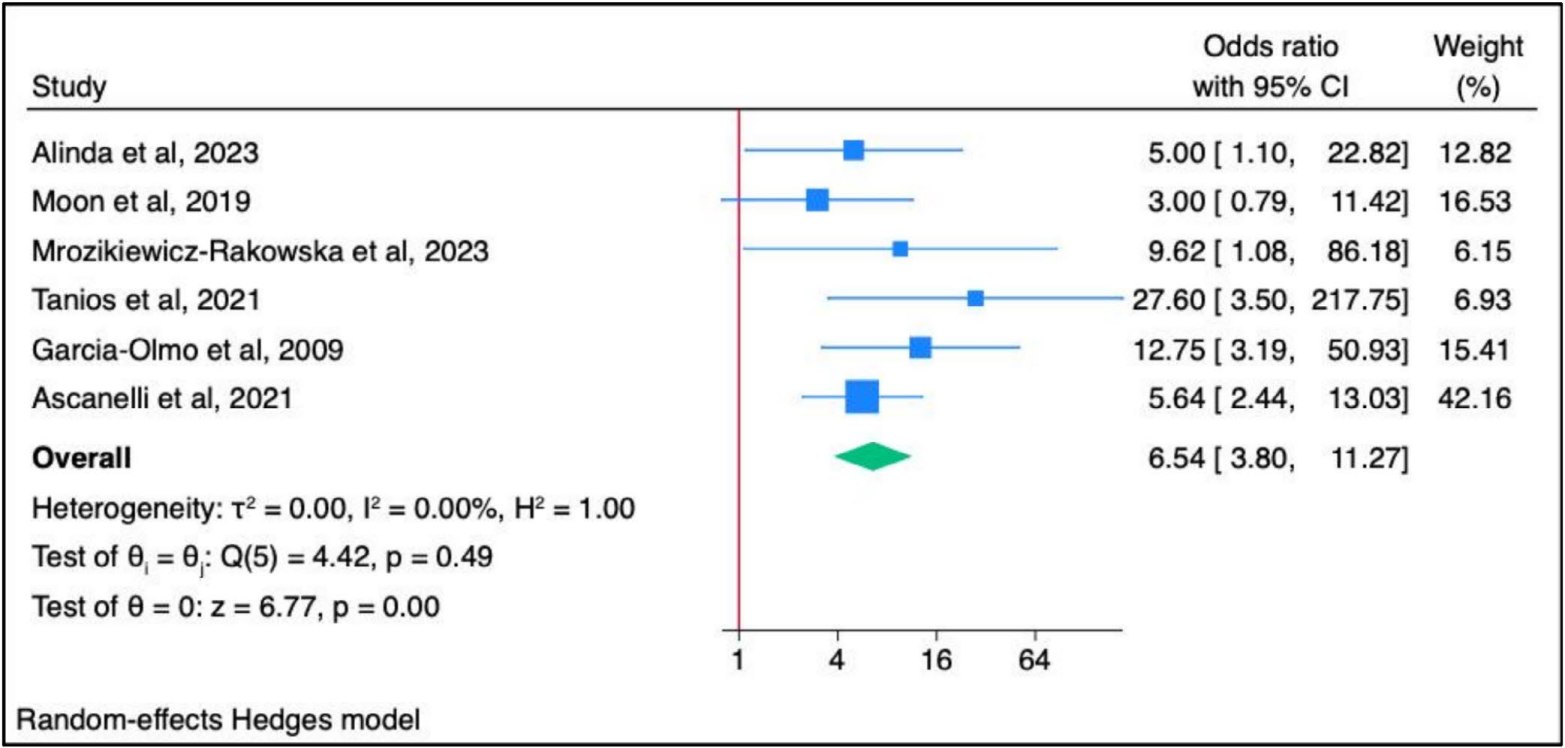
Forest plot displaying number of patients who achieved complete healing at short term follow‐up. CI: Confidence Interval. Statistical significance determined as *p* = < 0.05.

**FIGURE 3:**
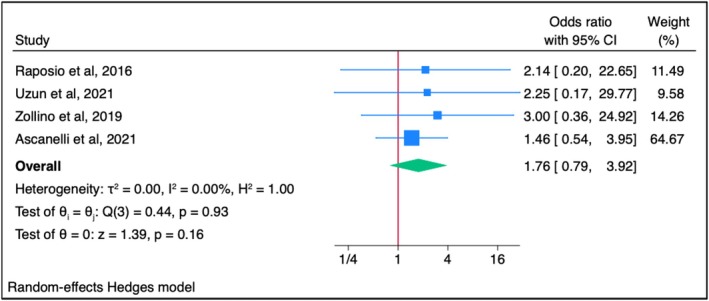
Forest plot showing number of patients who achieved complete healing at long‐term follow‐up. Long term outcomes considered when follow‐up time > 12 weeks. Statistical significance determined as *p* = < 0.05.

Eleven studies [14, 31, 32, 33, 34, 35, 36, 37, 38, 40, 41] measured the number of patients who achieved complete healing. Six studies performed short‐term follow‐up [14, 31, 34, 35, 37, 41] and four performed long‐term follow‐up [14, 36, 38, 40]. Ascanelli et al. [14] recorded numbers of healed participants at four different timepoints; short‐term follow‐up data was taken at 8 weeks, and long‐term was taken at 24 weeks. Studies which reported healing time < 12 weeks were considered short term, follow‐up time ranged from seven to 9 weeks. Pooled data from studies shows that patients treated with ADSCs were significantly more likely to heal in the short term (odds ratio (OR) 6.54, 95% confidence interval (CI) 3.80–11.27; *n* = 377 *I* ^2^ = 0.00% *p* = 0.00) (Figure 2). Pooled data from studies which reported rates of healing in the long‐term suggested that application of ADSCs made no significant improvement in likelihood to heal than those that didn't (OR 1.76, 95% CI 0.79–3.92; *n* = 192, *I* ^2^ = 0.00 *p* = 0.16) (Figure 3). Follow‐up time ranged from 12.3 to 24 weeks.

### 3.6 Time Taken to Heal

**FIGURE 4:**
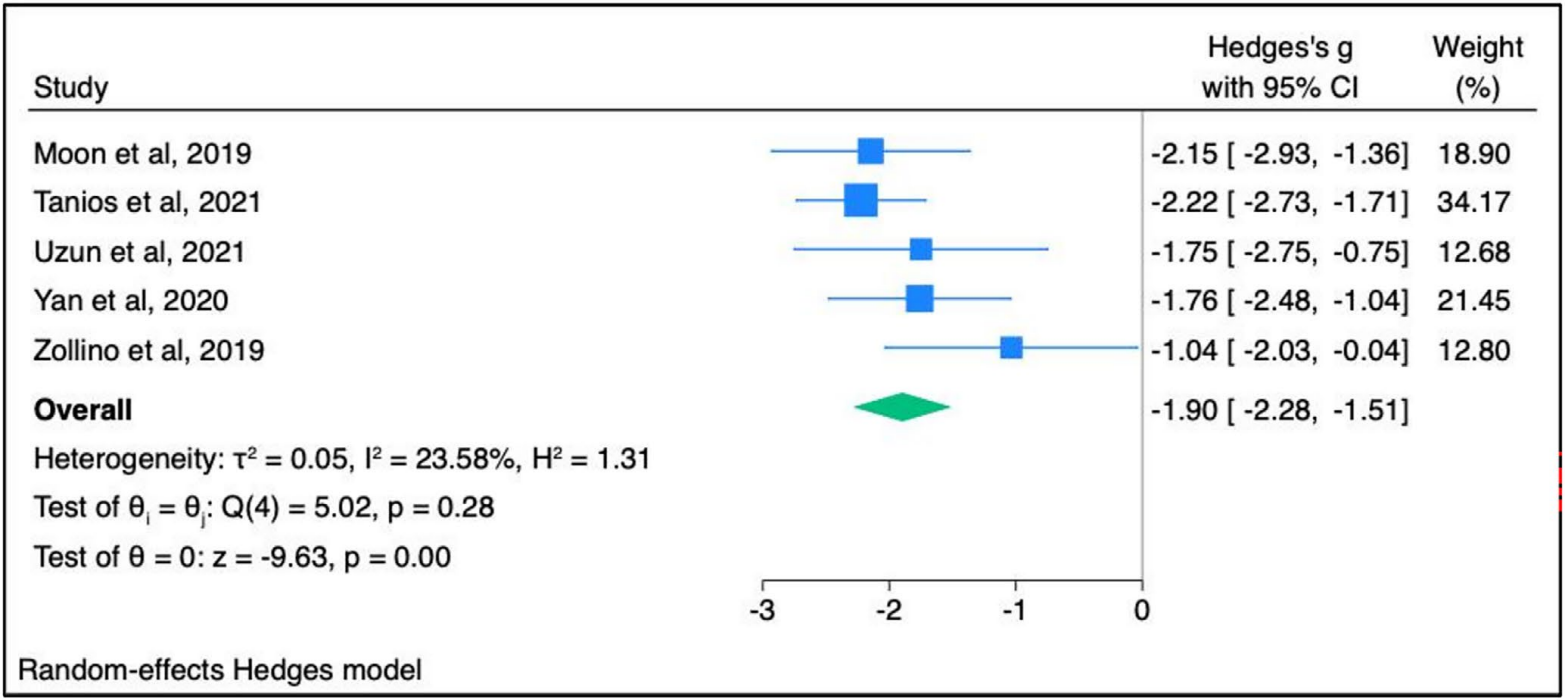
Forest plot showing time in days for wound to completely heal. Statistical significance determined as *p* = < 0.05.

Five studies provided data on healing time which was comparable and thus able to be included in the meta‐analysis [34, 37, 38, 39, 40]. Two studies [37, 40] provided their data as mean weeks ± SD; their data was converted to mean days ± SD before performing statistical analysis. Patients who had ADSC treatment took significantly less time to completely heal (mean difference −1.90, CI −2.28, −1.51; *n* = 210, *I* ^2^ = 23.58, *p* = 0.00) (Figure 4).

### 3.7 Patient Satisfaction

Not all studies reported patient satisfaction [31, 34, 36, 37, 39]. One study [33] mentioned only that the ADSC group experienced ‘less pain’ than the control group. Four studies [14, 38, 40, 41] did not find any statistically significant difference in patient satisfaction or pain scores between the two groups.

### 3.8 Adverse Outcomes

Six studies reported adverse events [34, 35, 36, 38, 40, 41], four were said to be unrelated to the ADSC treatment [34, 36, 38, 41]. In one study [40] a patient in the treatment group developed perilesional dermatitis which resolved spontaneously, and another [35] found no significant difference in occurrence of adverse events between the treatment and control groups.

## 4 Discussion

The key finding in this study is that patients treated with ADSCs are more likely to heal in the short‐term and heal faster than those treated with conventional therapies. The absence of a statistically significant difference in long‐term healing outcomes suggests that ADSCs may primarily accelerate the early phases of tissue repair rather than fundamentally alter the eventual likelihood of healing. Patients receiving conventional therapy may ultimately achieve similar healing outcomes given sufficient time. Alternatively, the lack of long‐term significance may reflect limited statistical power, variable follow‐up durations and the complex comorbidity profiles present within chronic wound populations. Despite the observed improvements in wound closure and healing time, studies reporting patient‐reported outcomes did not demonstrate statistically significant improvements in pain scores or patient satisfaction. This may indicate that accelerated wound healing does not necessarily translate into measurable short‐term improvements in perceived quality of life, particularly in patients with substantial comorbidity burdens and chronic disease. Elsharkawi et al. [42] published a meta‐analysis investigating the effect of ADSCs on diabetic ulcer healing. In contrast to this study, they found a significant increase in participants achieving full healing in the long term (at 12 months) compared to controls and a non‐significant increase in rate of healing. Elsharkawi et al. [42] included only diabetic ulcers; the meta‐analysis presented here includes a variety of wound types, although this is unlikely to fully account for the difference in outcomes. Studies on diabetic ulcers included in the meta‐analyses presented here [34, 37, 38] all demonstrated a statistically significant increase in healing achievement with ADSC administration in the short‐term (Figure 2). Elsharkawi et al. [42] included only three studies in their meta‐analysis, whereas this meta‐analysis included six studies in the short‐term healing achieved analysis, likely increasing the robustness of the results. Meta‐analyses of pre‐clinical ADSC animal studies report similar findings to this report, with ADSC groups having significantly faster healing times than controls [17, 43]. The methodology and patient populations between included studies were clinically heterogeneous, varying substantially in wound aetiology, comorbidity burden, ADSC source, preparation technique, concentration and administration route. Included wound types ranged from diabetic and venous ulcers to complex perianal fistulas, each with distinct underlying pathophysiology and healing trajectories. ADSC administration methods included intralesional injection and topical application, whilst studies utilised both autologous and allogeneic ADSCs. In addition, studies differed considerably in ADSC preparation and characterisation methods, with several failing to report exact cell concentrations or viability. Although statistical heterogeneity was low in pooled analyses, substantial clinical heterogeneity remained. Consequently, the pooled findings should be interpreted cautiously, and the observed benefits cannot be attributed solely to ADSCs with certainty. None of the reports in this study declared any serious adverse events which were directly related to ADSC administration, suggesting that it is a safe therapy.

### 4.1 Strengths

This review provides clinical evidence on the use of ADSCs to expedite the healing of chronic wounds from various aetiologies. The results presented here have the potential to inform clinical practise and guide future research into chronic wound treatment. This study included a variety of wounds and control treatments, in contrast to other meta‐analyses on a similar topic which focus on a singular wound type [42] or animal trials [17, 43]. This study, therefore, has more clinical relevance to the varied chronic wounds experienced by people in the UK today [3].

### 4.2 Limitations

The limitations of this study should be considered when interpreting these results. All included studies were considered to have moderate to high risk of bias, particularly due to lack of double‐blinding during wound assessment. Wound healing outcomes such as wound size reduction and complete healing contain subjective elements; therefore, absence of assessor blinding may have led to overestimation of the beneficial effects of ADSC therapy. In terms of study selection, excluding non‐English language and unpublished studies could have reduced the extent to which the results can be generalised. Screening and data extraction were not performed independently in duplicate, which may increase the risk of selection or extraction bias.

### 4.3 Future Direction

More high‐quality studies are needed in this field; all the studies included here had a moderate to high risk of bias. Clinical trials with double‐blinding, set concentrations of ADSCs and clear recording of comorbidities will be beneficial to confirm results seen in this study and determine a dose–response relationship. Few studies provided robust long‐term follow‐up data, and none comprehensively evaluated wound recurrence or durability of healing. Future clinical trials with larger sample sizes, longer follow‐up periods, standardised ADSC preparation protocols and rigorous blinding methods are required to determine whether ADSCs improve sustained healing outcomes in patients with chronic wounds and multiple comorbidities.

## 5 Conclusion

Evidence from this review suggests that ADSCs may improve healing in chronic wounds from a variety of aetiologies. ADSC therapy decreased the time it takes for wounds to heal and increased the likelihood of healing within the first 12 weeks of treatment. It is likely that patients treated with conventional therapies will achieve the same end outcomes as those with ADSC treatment. Further clinical trials investigating ADSC application will help elucidate their full potential in expediting healing.

## Funding

The authors have nothing to report.

## Ethics Statement

This meta‐analysis does not require ethics approval; the data included in this report has been derived from studies whose original authors have obtained ethics approval.

## Conflicts of Interest

The authors declare no conflicts of interest.

## Data Availability

The data that support the findings of this study are available from the corresponding author upon reasonable request.

## Appendix A

### See Table A1

**TABLE A1:** Literature searches.

Bibliographic databases		
Initial searches		
Database	Date searched	Number of records
MEDLINE All (via Ovid)	09/10/24	985
Embase (via Ovid)	09/10/24	189
Web of Science	10/10/24	1971
PubMed	10/10/24	41
Total number of records retrieved: 3186 Duplicates removed (Rayyan + EndNote): 689 Final number for screening: 2497		

Updated searches		
Database	Date searched	Number of records
MEDLINE All and Embase (via Ovid)	02/06/25	250
Web of Science	04/06/25	124
PubMed	04/06/25	162
Total number of records retrieved: 536 Duplicates removed (Rayyan): 137 Final number for screening: 399		

Combined searches
Due to errors in limiting searches by date, some articles appeared in both first and second searches, leading to a higher number of duplicates when considered together. Total number of records retrieved: 3722 Duplicates removed (Rayyan): 1024 Final number for screening: 2698

## Appendix B OVID Search Used for EMBASE and Medline.

Ovid MEDLINE(R) ALL <1946 to June 2, 2025>.

1. stem cells.mp. or Stem Cells/
2. Cell differentiation/or mesenchymal stem cells.mp. or Mesenchymal Stem Cells/
3. stem cell transplantation or mesenchymal stem cell transplantation or tissue transplantation/
4. Adipose.mp.
5. ADSC.mp.
6. Adipose‐derived stem cells.mp.
7. Wound Healing/or chronic wounds.mp
8. nerve regeneration or wound healing or tissue survival
9. wound infection or surgical wound infection/
10. ‘wounds and injuries’/or arm injuries/or burns/or cold injury/or crush injuries/or electric injuries/or frostbite/or hand injuries/or lacerations/or leg injuries/or radiation injuries/or soft tissue injuries/or wounds, nonpenetrating/or wounds, penetrating/
11. Diabetic Foot/
12. Skin Ulcer/or Leg Ulcer/or Varicose Ulcer/or Pressure Ulcer/or ulcer.mp. or Ulcer/or Foot Ulcer/
13. 1 or 2 or 3
14. 4 and 13
15. 5 or 6 or 14
16. 7 or 8 or 9 or 10 or 11 or 12
17. 15 and 16
18. exp. animals/not humans.sh.
19. 17 not 18

## Appendix C

### See Table C1

**TABLE C1:** Patient characteristics by study.

Study	Participants (*n*)		Treatment	Control	Gender (*n*)	Age (Mean ± SD)	
	ADSC	Control			F/M	ADSC	Control
Alinda et al. [31]	16	16	Topical ADSC	Framycetin gauze	13/19	45.75 ± 4.97	45.19 ± 7.22
Ascanelli et al. [14]	58	58	Surgery + ADSC injection	Surgery	45/71	50.41 ± 14.2	50.41 ± 14.8
Garcia‐Olmo et al. [41]	24	25	Surgery + ADSC injection	Surgery	25/24	42.64 ± 10.93	43.99 ± 8.97
Larsen et al. [32]	1		Topical ADSC	Iodine dressing	0/1	63	
Marino et al. [33]	10	10	ADSC injection	Dressings	6/14	65.8 ± 3.08^a^	64.8 ± 3.85^a^
Moon et al. [34]	22	17	ADSC dressing	Silicone dressing	12/27	59.9 ± 13.3	68.4 ± 9.9
Mrozikiewicz‐Rakowska et al. [35]	23	23	Fibrin Gel + ADSC	Fibrin Gel	9/37	68.4 ± 9.9	61.7 ± 7.5
Raposio et al. [36]	16	24	ADSC + PRP injection	PRP	19/21	70.76	74.6
Tanios et al. [37]	50 [47]	50 [48]	ADSC injection	Betadine ointment	47/53	48.12 ± 14.58	48.04 ± 10.56
Uzun et al. [38]	10	10	ADSC injection	Dressings	10/10	57.5 ± 8.4	57.5 ± 8.4
Yan et al. [39]	20	20	ADSC injection	Dressings	19/21	42.9 ± 19.5	48.2 ± 14.9
Zollino et al. [40]	8	8	ADSC injection	Dressings	6/10	75 ± 6.7	69 ± 12.8

*Note:* Studies are ordered alphabetically by first author.

Abbreviations: ADSC: Adipose‐Derived Stem Cells, F: Female, M: Male, NI: No Information, PRP: Platelet Rich Plasma, SD: Standard Deviation.

^a^ Calculated by the researcher. Numbers in square brackets represent patient numbers after losses to follow‐up.

## Appendix D

### See Table D1

**TABLE D1:** Patient characteristics by outcome studied.

		Healing achieved—short term [14, 31, 34, 35, 37, 41]	Healing achieved—long term [14, 36, 38, 40]	Time taken to heal [34, 37, 38, 39, 40]	Total included [14, 31, 34, 35, 36, 37, 38, 39, 40, 41]	Total [14, 31, 32, 33, 34, 35, 36, 37, 38, 39, 40, 41]
Wound type number (%)	Diabetic ulcer	162 (42.4)	20 (10.4)	136 (63.3)	182 (36.5)	182 (35.1)
	Venous ulcer	1 (0.3)	11 (5.7)	12 (5.6)	12 (2.4)	13 (2.5)
	Arterial ulcer	0 (0.0)	0 (0.0)	0 (0.0)	0 (0.0)	20 (3.9)
	Leprosy ulcer	32 (8.4)	0 (0.0)	0 (0.0)	32 (6.4)	32 (6.2)
	Fistula	165 (43.8)	116 (60.4)	0 (0.0)	165 (33.1)	165 (31.8)
	Trophic ulcer	20 (5.3)	0 (0.0)	20 (9.3)	20 (4.0)	20 (3.9)
	Mixed/not specified	2 (0.5)	45 (23.4)	47 (21.9)	87 (17.5)	87 (16.8)
Comorbidities number (%)	Diabetes mellitus	173 (45.3)	36 (18.8)	141 (65.6)	216 (42.0)	217 (41.8)
	CVD	43 (11.3)	13 (6.8)	47 (21.9)	76 (14.8)	76 (34.7)
	Leprosy	32 (8.5)	0 (0.0)	0 (0.0)	32 (6.2)	32 (14.6)
	Crohn's	130 (34.0)	116 (60.4)	0 (0.0)	130 (25.0)	130 (59.4)
	Other	55 (14.4)	2 (1.0)	19 (8.8)	63 (12.1)	31 (14.2)

*Note:* Data is presented as number of patients with condition and percentage. Patients with characteristics such as ankle‐brachial pressure index < 0.8, ischaemic heart disease, and coronary artery disease have been listed under CVD. Percentages represent the percentage of patients with that comorbidity within that outcome group. The sum of patients with a comorbidity does not equal the total number of patients in that outcome due to some patients having multiple comorbidities and some studies not publishing comorbidity data. Healing Achieved—Short Term and Time Taken to Heal both include data from Tanios et al. [37] who lost five patients to follow‐up. Their wound types, therefore, total five more than the number of patients included in the final study.

Abbreviation: CVD: Cardiovascular disease.

## Appendix E

### See Table E1

**TABLE E1:** ADSC characteristics by study.

Study	Type	Preparation	Delivery method	Characterisation	Volume applied (mL)	Concentration (cells per mL)
Alinda et al. [31]	NI	Isolation of ADSC from lipoaspirate, then in vitro expansion	Topical	NI	NI	NI
Ascanelli et al. [14]	Autologous	Lipoaspirate centrifuged, then returned to patient—sample taken for characterisation	Injection	NI	NI	NI
Garcia‐Olmo et al. [41]	Autologous	Isolation of ADSC from lipoaspirate, then in vitro expansion	Injection	Immunopheno‐typing	NI	3–30 × 10^6^
Larsen et al. [32]	Autologous	NI	Topical	Flow cytometry	NI	NI
Marino et al. [33]	Autologous	Isolation of ADSC from lipoaspirate, then in vitro expansion	Injection	Flow cytometry	3	3 × 10^5^
Moon et al. [34]	Allogenic	Isolation of ADSC from lipoaspirate, then in vitro expansion	Topical	Immunopheno‐typing	NI	NI
Mrozikiewicz‐Rakowska et al. [35]	Allogenic	Isolation of ADSC from lipoaspirate, then in vitro expansion	Topical	Flow cytometry	1	2.5 × 10^6^
Raposio et al. [36]	Autologous	Lipoaspirate centrifuged, then returned to patient	Injection	Centrifugation alone	NI	Nil
Tanios et al. [37]	Autologous	Isolation of ADSC from lipoaspirate, then in vitro expansion	Injection	Flow cytometry	NI	NI
Uzun et al. [38]	Allogenic	Isolation of ADSC from lipoaspirate, then in vitro expansion	Injection	Flow cytometry	NI	6 × 10^6^*
Yan et al. [39]	Autologous	Isolation of ADSC from lipoaspirate	Injection	Centrifugation	10	5 × 10^6^
Zollino et al. [40]	Autologous	Lipoaspirate centrifuged, then returned to patient—sample taken for characterisation	Injection	Flow cytometry	NI	NI

*Note:* Studies are ordered alphabetically by first author. Concentration presented as cells per mL, with the exception of data marked with an asterisk which did not provide a volume.

Abbreviations: mL: millilitres, NI: No information.

## References

[1] Trade DfI , Advanced Wound Care: Develop New Treatments in the UK (Department for International Trade, 2018), https://www.gov.uk/government/publications/advanced‐wound‐care‐develop‐treatments‐in‐the‐uk/advanced‐wound‐care‐develop‐new‐treatments‐in‐the‐uk.

[2] A. Gosain and D. P. LA , “Aging and Wound Healing,” World Journal of Surgery 28, no. 3 (2004): 321–326. 14961191 10.1007/s00268-003-7397-6

[3] W. U. Hassan , U. Greiser , and W. Wang , “Role of Adipose‐Derived Stem Cells in Wound Healing,” Wound Repair and Regeneration 22, no. 3 (2014): 313–325. 24844331 10.1111/wrr.12173

[4] Care IfQaEiH , Chronic Wounds: Learn More—What Are the Treatment Options for Chronic Wounds (Institute for Quality and Efficiency in Health Care, 2022), https://www.ncbi.nlm.nih.gov/books/NBK326436/.

[5] N. Bertozzi , F. Simonacci , M. P. Grieco , E. Grignaffini , and E. Raposio , “The Biological and Clinical Basis for the Use of Adipose‐Derived Stem Cells in the Field of Wound Healing,” Annals of Medicine and Surgery 20 (2017): 41–48. 28702186 10.1016/j.amsu.2017.06.058 PMC5491486

[6] M. Olsson , K. Järbrink , U. Divakar , et al., “The Humanistic and Economic Burden of Chronic Wounds: A Systematic Review,” Wound Repair and Regeneration 27, no. 1 (2019): 114–125. 30362646 10.1111/wrr.12683

[7] CKS N , Leg Ulcer—Venous (NICE CKS, 2024), https://cks.nice.org.uk/topics/leg‐ulcer‐venous/.

[8] NICE , “Chronic Wounds: Advanced Wound Dressings and Antimicrobial Dressings,” 2016, https://www.nice.org.uk/advice/esmpb2/resources/chronic‐wounds‐advanced‐wound‐dressings‐and‐antimicrobial‐dressingspdf‐1502609570376901.

[9] Y.‐G. Koh and Y.‐J. Choi , “Infrapatellar Fat Pad‐Derived Mesenchymal Stem Cell Therapy for Knee Osteoarthritis,” Knee 19, no. 6 (2012): 902–907. 22583627 10.1016/j.knee.2012.04.001

[10] C. H. Jo , Y. G. Lee , W. H. Shin , et al., “Intra‐Articular Injection of Mesenchymal Stem Cells for the Treatment of Osteoarthritis of the Knee: A Proof‐Of‐Concept Clinical Trial,” Stem Cells 32, no. 5 (2014): 1254–1266. 24449146 10.1002/stem.1634

[11] Y. Cai , F. Zhang , J. Feng , et al., “Long‐Term Follow‐Up and Exploration of the Mechanism of Stromal Vascular Fraction Gel in Chronic Wounds,” Stem Cell Research & Therapy 14, no. 1 (2023): 163. 37337292 10.1186/s13287-023-03389-2 PMC10280847

[12] C. Long , J. Wang , W. Gan , X. Qin , R. Yang , and X. Chen , “Therapeutic Potential of Exosomes From Adipose‐Derived Stem Cells in Chronic Wound Healing,” Frontiers in Surgery 9 (2022): 1030288. 36248361 10.3389/fsurg.2022.1030288 PMC9561814

[13] N. Hu , Z. Cai , X. Jiang , et al., “Hypoxia‐Pretreated ADSC‐Derived Exosome‐Embedded Hydrogels Promote Angiogenesis and Accelerate Diabetic Wound Healing,” Acta Biomaterialia 157 (2023): 175–186. 36503078 10.1016/j.actbio.2022.11.057

[14] S. Ascanelli , P. Zamboni , D. Campioni , et al., “Efficacy and Safety of Treatment of Complex Idiopathic Fistula‐In‐Ano Using Autologous Centrifuged Adipose Tissue Containing Progenitor Cells: A Randomized Controlled Trial,” Diseases of the Colon and Rectum 64, no. 10 (2021): 1276–1285. 34016825 10.1097/DCR.0000000000001924

[15] Y. H. K. Cao , X. Ma , Y. Zhang , J. Huang , and X. Long , “Autologous Fat or Adipose‐Derived Stem Cell Grafting in Systemic Sclerosis Treatment: A Systematic Review and Meta‐Analysis,” Clinical and Experimental Rheumatology 41, no. 8 (2023): 1659–1669. 37382451 10.55563/clinexprheumatol/ycy3k7

[16] S. Lee , D. S. Chae , B. W. Song , et al., “ADSC‐Based Cell Therapies for Musculoskeletal Disorders: A Review of Recent Clinical Trials,” International Journal of Molecular Sciences 22, no. 19 (2021): 10586. 34638927 10.3390/ijms221910586 PMC8508846

[17] J. T. Wei , T. He , K. Shen , Z. G. Xu , J. T. Han , and X. K. Yang , “Adipose Stem Cell‐Derived Exosomes in the Treatment of Wound Healing in Preclinical Animal Models: A Meta‐Analysis,” Burns & Trauma 12 (2024): tkae025. 39099759 10.1093/burnst/tkae025 PMC11298109

[18] M. H. Carstens , F. J. Quintana , S. T. Calderwood , et al., “Treatment of Chronic Diabetic Foot Ulcers With Adipose‐Derived Stromal Vascular Fraction Cell Injections: Safety and Evidence of Efficacy at 1 Year,” Stem Cells Translational Medicine 10, no. 8 (2021): 1138–1147. 33826245 10.1002/sctm.20-0497 PMC8284780

[19] Y. B. Cho , K. J. Park , S. N. Yoon , et al., “Long‐Term Results of Adipose‐Derived Stem Cell Therapy for the Treatment of Crohn's Fistula,” Stem Cells Translational Medicine 4, no. 5 (2015): 532–537. 25829404 10.5966/sctm.2014-0199 PMC4414218

[20] M. J. Page , D. Moher , P. M. Bossuyt , et al., “PRISMA 2020 Explanation and Elaboration: Updated Guidance and Exemplars for Reporting Systematic Reviews,” BMJ 372 (2021): n160. 33781993 10.1136/bmj.n160 PMC8005925

[21] Team TE , Endnote, 21st ed. (Clarivate, 2013).

[22] W. M. Bramer , D. Giustini , G. B. de Jonge , L. Holland , and T. Bekhuis , “De‐Duplication of Database Search Results for Systematic Reviews in EndNote,” Journal of the Medical Library Association 104, no. 3 (2016): 240–243. 27366130 10.3163/1536-5050.104.3.014 PMC4915647

[23] O. H. H. Mourad , Z. Fedorowicz , and A. Elmagarmid , “Rayyan—A Web and Mobile App for Systematic Reviews,” Systematic Reviews 5, no. 1 (2016): 210. 27919275 10.1186/s13643-016-0384-4 PMC5139140

[24] Microsoft Corporation , *M*icrosoft Excel Computer Software 16.89.1 (Microsoft Corporation, 2018).

[25] J. P. T. Higgins , J. Thomas , J. Chandler , et al., Cochrane Handbook for Systematic Reviews of Interventions [eBook] version 6.5 (updated August 2024), www.training.cochrane.org/handbook.

[26] J. A. Sterne , M. A. Hernán , B. C. Reeves , et al., “ROBINS‐I: A Tool for Assessing Risk of Bias in Non‐Randomised Studies of Interventions,” BMJ 355 (2016): i4919. 27733354 10.1136/bmj.i4919 PMC5062054

[27] S. JAC , J. Savović , M. J. Page , et al., “RoB 2: A Revised Tool for Assessing Risk of Bias in Randomised Trials,” BMJ 366 (2019): l4898. 31462531 10.1136/bmj.l4898

[28] J. L. Brozek , E. A. Akl , P. Alonso‐Coello , et al., “Grading Quality of Evidence and Strength of Recommendations in Clinical Practice Guidelines. Part 1 of 3. An Overview of the GRADE Approach and Grading Quality of Evidence About Interventions,” Allergy 64, no. 5 (2009): 669–677. 19210357 10.1111/j.1398-9995.2009.01973.x

[29] L. V. Hedges and J. L. Vevea , “Fixed‐ and Random‐Effects Models in Meta‐Analysis,” Psychological Methods 3, no. 4 (1998): 486–504.

[30] StataCorp , Stata Statistical Software: Release 18 (StataCorp LLC, 2023).

[31] M. D. Alinda , P. M. Christopher , M. Y. Listiawan , et al., “The Efficacy of Topical Adipose Mesenchymal Stem Cell‐Conditioned Medium Versus Framycetin Gauze Dressing in Chronic Plantar Ulcer of Leprosy: A Randomized Controlled Trial,” Indian Journal of Dermatology, Venereology and Leprology 89, no. 5 (2023): 656–664. 36688887 10.25259/IJDVL_784_2021

[32] L. Larsen , C. N. Tchanque‐Fossuo , F. Gorouhi , et al., “Combination Therapy of Autologous Adipose Mesenchymal Stem Cell‐Enriched, High‐Density Lipoaspirate and Topical Timolol for Healing Chronic Wounds,” Journal of Tissue Engineering and Regenerative Medicine 12, no. 1 (2018): 186–190. 27943665 10.1002/term.2390

[33] G. Marino , M. Moraci , E. Armenia , et al., “Therapy With Autologous Adipose‐Derived Regenerative Cells for the Care of Chronic Ulcer of Lower Limbs in Patients With Peripheral Arterial Disease,” Journal of Surgical Research 185, no. 1 (2013): 36–44. 23773718 10.1016/j.jss.2013.05.024

[34] K.‐C. Moon , H.‐S. Suh , K.‐B. Kim , et al., “Potential of Allogeneic Adipose‐Derived Stem Cell‐Hydrogel Complex for Treating Diabetic Foot Ulcers,” Diabetes 68, no. 4 (2019): 837–846. 30679183 10.2337/db18-0699

[35] B. Mrozikiewicz‐Rakowska , I. Szablowska‐Gadomska , D. Cysewski , et al., “Allogenic Adipose‐Derived Stem Cells in Diabetic Foot Ulcer Treatment: Clinical Effectiveness, Safety, Survival in the Wound Site, and Proteomic Impact,” International Journal of Molecular Sciences 24, no. 2 (2023): 1472. 36674989 10.3390/ijms24021472 PMC9864558

[36] E. Raposio , N. Bertozzi , S. Bonomini , et al., “Adipose‐Derived Stem Cells Added to Platelet‐Rich Plasma for Chronic Skin Ulcer Therapy,” Wounds: a Compendium of Clinical Research and Practice 28, no. 4 (2016): 126–131. 27071140

[37] E. Tanios , T. M. Ahmed , E. A. Shafik , et al., “Efficacy of Adipose‐Derived Stromal Vascular Fraction Cells in the Management of Chronic Ulcers: A Randomized Clinical Trial,” Regenerative Medicine 16, no. 11 (2021): 975–988. 34596433 10.2217/rme-2020-0207

[38] E. Uzun , A. Guney , Z. B. Gonen , et al., “Intralesional Allogeneic Adipose‐Derived Stem Cells Application in Chronic Diabetic Foot Ulcer: Phase I/2 Safety Study,” Foot and Ankle Surgery: Official Journal of the European Society of Foot and Ankle Surgeons 27, no. 6 (2021): 636–642. 32826167 10.1016/j.fas.2020.08.002

[39] X. Yan , Y. A. Jiang , Y. Xu , and Q. Tan , “The Effect of Adipose‐Derived Stem Cells in Healing Refractory Wounds Based on Clinical Outcomes,” All Life 13, no. 1 (2020): 433–439.

[40] I. Zollino , D. Campioni , M. G. Sibilla , M. Tessari , A. M. Malagoni , and P. Zamboni , “A Phase II Randomized Clinical Trial for the Treatment of Recalcitrant Chronic Leg Ulcers Using Centrifuged Adipose Tissue Containing Progenitor Cells,” Cytotherapy 21, no. 2 (2019): 200–211. 30583949 10.1016/j.jcyt.2018.10.012

[41] D. Garcia‐Olmo , D. Herreros , I. Pascual , et al., “Expanded Adipose‐Derived Stem Cells for the Treatment of Complex Perianal Fistula: A Phase II Clinical Trial,” Diseases of the Colon and Rectum 52, no. 1 (2009): 79–86. 19273960 10.1007/DCR.0b013e3181973487

[42] M. Elsharkawi , B. Ghoneim , M. O'Sullivan , et al., “Role of Adipose Derived Stem Cells in Patients With Diabetic Foot Ulcers: Systematic Review and Meta‐Analysis of Randomised Controlled Trials,” International Journal of Lower Extremity Wounds 5, no. 3 (2023): 542–549. 10.1177/15347346231174554 37170536

[43] T. Yuan , L. Meijia , C. Xinyao , C. Xinyue , and H. Lijun , “Exosome Derived From Human Adipose‐Derived Stem Cell Improve Wound Healing Quality: A Systematic Review and Meta‐Analysis of Preclinical Animal Studies,” International Wound Journal 20, no. 6 (2023): 2424–2439. 37102269 10.1111/iwj.14081 PMC10333007

